# Main pancreatic duct dilatation and pancreatic cysts in relatives and spouses of patients with pancreatic cancer

**DOI:** 10.1371/journal.pone.0280403

**Published:** 2023-01-11

**Authors:** Kenji Ikezawa, Sachiko Tanaka, Junko Fukuda, Miho Nakao, Yoko Nakano, Mayumi Chagi, Hiromi Yamanaka, Kazuyoshi Ohkawa

**Affiliations:** 1 Department of Hepatobiliary and Pancreatic Oncology, Osaka International Cancer Institute, Osaka, Japan; 2 Osaka International Cancer Institute, Osaka, Japan; 3 Department of Clinical Laboratory, Osaka International Cancer Institute, Osaka, Japan; University of Nebraska Medical Center, UNITED STATES

## Abstract

Although main pancreatic duct dilatation and pancreatic cysts are risk factors for developing pancreatic cancer, limited data exist regarding these findings in relatives and spouses of pancreatic cancer patients. The frequency of these findings was examined using long-term follow-up data and transabdominal ultrasonography focusing on the pancreas. We prospectively enrolled 184 relatives and spouses of pancreatic cancer patients and performed special pancreatic ultrasonography to detect main pancreatic duct dilatation and pancreatic cysts. First-degree relatives (148 participants) of patients with pancreatic cancer were significantly younger than the spouses (36 participants; 41 vs. 65 years old). The frequency of ultrasonographic findings was significantly different between the relative (8.8%) and spouse (33.3%) groups. Main pancreatic duct dilatation and pancreatic cysts were observed in seven (4.7%) and seven (4.7%) participants in the relative group, and in nine (25.0%) and five (13.9%) participants in the spouse group, respectively. On multivariate analysis, age was an independent risk factor for the ultrasonographic findings. The frequency of ultrasonographic findings was significantly higher in spouses than in first-degree relatives of patients with pancreatic cancer and was strongly influenced by the age gap between the groups. Main pancreatic duct dilatation was frequently observed, especially in the spouse group.

## Introduction

Approximately 80% of patients with pancreatic cancer (PC) are diagnosed with metastatic or locally advanced disease [1]. Although recent progress in chemotherapy and chemoradiotherapy has improved prognosis to some extent, patients with PC still have a poor prognosis, with a 5-year overall survival rate of 9% [2–5]. Because it is difficult to detect PC at an early stage, surveillance with imaging modalities for the appropriate target is important [6–9]. Recently, it has been demonstrated that the surveillance of high-risk individuals with genetic factors of family history is an efficient strategy for detecting resectable PC [6].

There are several risk factors for PC, including diabetes mellitus, chronic pancreatitis, and smoking history [10]. Family history is also associated with an increased risk of developing PC [11–14]. Klein et al. reported that individuals with a strong family history of PC had a significantly increased risk of PC, while spouses and other genetically unrelated relatives did not have a significantly increased risk of PC (standardized incidence ratio, 2.4; 95% confidence interval [CI], 0.06–13.5) [11]. Although environmental factors may affect the risk of PC and pancreatic diseases, there have been few studies on this among spouses of patients with PC [11, 15].

Conventional transabdominal ultrasonography is an efficient method for screening pancreatic diseases owing to its minimal invasiveness. However, the ability of conventional transabdominal ultrasonography to detect PC is affected by obesity and the presence of gastric gas [16]. To overcome these difficulties, we developed a special ultrasound method focusing on the pancreas (special pancreatic ultrasonography), which is performed with the patient in a sitting position following liquid ingestion to decrease the effect of gastric gas [17]. Special pancreatic ultrasonography demonstrated superiority over conventional transabdominal ultrasonography in detecting pancreatic cysts [16]. Recently, we found that early detection of PC and favorable prognoses were achieved by surveillance, mainly with special pancreatic ultrasonography, in high-risk individuals with pancreatic cysts and/or dilatation of the main pancreatic duct (MPD) (defined as an MPD diameter of ≥2.5 mm) [7].

Based on a prospective follow-up, we previously reported that MPD dilatation and the presence of pancreatic cysts are risk factors for developing PC [18]. The hazard ratio for PC was as high as 27.5 in individuals who had both MPD dilatation and pancreatic cysts. However, limited data exist regarding these findings in relatives and spouses of patients with PC. In this study, we examined the frequency of having MPD dilatation and pancreatic cysts in these individuals, using special pancreatic ultrasonography and long-term follow-up data.

## Methods

### Study design and participants

Between April 2009 and March 2013, a total of 184 participants were prospectively enrolled in this study. Written informed consent was obtained from all the participants. The inclusion criteria were as follows: relatives or spouses of patients with pathologically proven PC, individuals aged 20–79 years, and individuals with no active or recent malignancies of other organs.

Data on age, sex, family history of PC, body mass index (BMI), and history of smoking, drinking, diabetes mellitus, and pancreatitis were collected for each participant.

At enrollment, special pancreatic ultrasonography was performed for all participants, and ultrasonographic findings, including dilatation of the MPD (defined as ≥2.5 mm in diameter) and pancreatic cysts (defined as ≥5 mm in diameter), were collected. The MPD diameter was measured in the body of the pancreas before and after the ingestion of liquid, and the larger size was adopted as the finding. The major axis diameter of the cyst was used as the cyst size. Special pancreatic ultrasonography was performed primarily by sonographers with ≥7 years of experience according to a previously published protocol [7, 16, 17, 19]. At least 12 standard images of the pancreas were recorded before and after liquid ingestion based on our manual of procedures. To allow the participant to maintain the position easily, an examination table with an adjustable backrest was used. After screening the pancreas by changing the patient’s position, the liquid-filled stomach method was used to improve visualization of the pancreas, especially the pancreatic body and tail. Commercially available black tea with milk or green tea (350 mL) was primarily used in our hospital.

The clinical courses of the participants were followed up using questionnaires between November 2020 and February 2021 and/or medical records at our institution, if applicable. This study was approved by the Institutional Review Board of the Osaka International Cancer Institute (No. 20164) and was performed in accordance with the Declaration of Helsinki.

### Statistical analysis

Categorical variables are presented as percentages and continuous variables as medians and ranges. Participant characteristics were compared using the chi-square test or Fisher’s exact test for categorical variables, or the Mann–Whitney U-test for continuous variables.

To examine factors associated with ultrasonographic findings, odds ratios (ORs) and 95% CIs were calculated using univariate and multivariate logistic regression analyses. Factors with a P-value <0.10 on univariate analysis were entered into the multivariate analyses. Statistical analyses were performed using EZR, a graphical interface of the R Commander software package for Windows (version 1.50) [20]. A P-value <0.05 was considered statistically significant.

## Results

### Participant characteristics

The characteristics of the 184 participants included in this study are summarized in Table 1. Sixty-four participants (34.8%) were men, and the median age was 44 years (range, 20–76 years). The median BMI was 21.6 kg/m^2^ (range, 16.4–31.7 kg/m^2^).

**Table 1:** Background of participants.

		Total	Relatives	Spouses	P-value
		(n = 184)	(n = 148)	(n = 36)	
Blood relationship to patients with PC (children/siblings/parents)		—	102/42/4	—	
The number of first-degree relatives of the participant with PC (0/1/2/3)		35/140/7/2	0/139/7/2	35/1/0/0	**<0.001** (0 vs ≥1)
Age, median (range), years		44 (20–76)	41 (20–73)	65 (40–76)	**<0.001**
Sex	Male, n (%)	64 (34.8)	58 (39.2)	6 (16.7)	**0.019**
	Female, n (%)	120 (65.2)	90 (60.8)	30 (83.3)	
BMI, median (range), kg/m^2^		21.6 (16.4–31.7)	21.7 (16.4–31.7)	21.4 (16.8–29.7)	0.623
History of smoking (current/past/nonsmoker)		19/33/132	18/30/100	1/3/32	**0.012** (current/past vs nonsmoker)
History of drinking (current/past/non-drinker)		99/3/82	87/0/61	12/3/21	0.096 (current/past vs nondrinker)
History of diabetes mellitus (yes/no)		8/176	5/143	3/33	0.190
History of pancreatitis (yes/no)		6/178	5/143	1/35	1

PC, pancreatic cancer; BMI, body mass index

Baseline characteristics were compared between relatives and spouses of patients with PC (Table 1). In the relative group, all participants had a family history of PC in first-degree relatives (one in 139 participants, two in seven participants, and three in two participants). In the spouse group, only one participant (2.8%) was a first-degree relative of a patient with PC. Relatives were significantly younger than spouses of patients with PC (41 vs. 65 years old; P < 0.001). The proportion of women was significantly smaller in the relative group than in the spouse group (60.8% vs. 83.3%; P = 0.001). There were no significant differences in BMI, history of smoking, drinking, diabetes, or pancreatitis between the two groups.

### Factors associated with ultrasonographic findings

Relevant ultrasonographic findings were found in 13.6% (25/184) of participants (Table 2).

**Table 2:** Ultrasonographic findings of participants.

	Total	Relatives	Spouses	P-value
	(n = 184)	(n = 148)	(n = 36)	
**Ultrasonographic findings**	**13.6% (25/184)**	**8.8% (13/148)**	**33.3% (12/36)**	**<0.001**
MPD dilatation	13	6	7	
Pancreatic cysts	9	6	3	
Presence of both MPD dilatation and pancreatic cysts	3	1	2	

MPD dilatation was defined as dilatation ≥2.5 mm in diameter, and pancreatic cysts as ≥5 mm in diameter.

MPD, main pancreatic duct

A single finding was observed in 22 participants (MPD dilatation, 13; pancreatic cysts, nine). Both MPD dilatation and pancreatic cysts were detected in three participants. The frequency of ultrasonographic findings was significantly different between the relative group (8.8%, 13/148) and the spouse group (33.3%, 12/36) (P = 0.001). Among the 148 relatives, MPD dilatation and pancreatic cysts were found in seven (4.7%) and seven (4.7%) participants, respectively. Among the 36 spouses, MPD dilatation and pancreatic cysts were found in nine (25.0%) and five (13.9%) participants, respectively.

The univariate logistic regression analysis revealed that age and being a spouse were significant risk factors for ultrasonographic findings (Table 3). Furthermore, the multivariate analysis identified age as an independent risk factor for ultrasonographic findings (OR, 6.740; 95% CI, 1.820–24.900; P = 0.004) (Table 3). Spouses tended to correlate with a higher risk of ultrasonographic findings, although the difference was not statistically significant (OR, 2.590; 95% CI, 0.993–6.750; P = 0.052).

**Table 3:** Factors associated with ultrasonographic findings.

Factor		Univariate		Multivariate	
		Odds ratio (95% CI)	P-value	Odds ratio (95% CI)	P-value
Age	<45 years	1		1	
	≥45 years	**9.570 (2.750–33.300)**	**<0.001**	**6.740 (1.820–24.900)**	**0.004**
Sex	Female	1			
	Male	0.866 (0.352–2.130)	0.753		
BMI	<21 kg/m^2^	1			
	≥21 kg/m^2^	1.010 (0.427–2.390)	0.981		
History of smoking	No	1			
	Yes	0.596 (0.211–1.680)	0.328		
History of drinking	No	1			
	Yes	0.587 (0.251–1.370)	0.219		
History of diabetes mellitus	No	1			
	Yes	2.22 (0.422–11.700)	0.347		
Relationship	Relatives	1		1	
	Spouses	**5.190 (2.120–12.700)**	**<0.001**	2.590 (0.993–6.750)	0.052

MPD dilatation was defined as ≥2.5 mm in diameter, and pancreatic cysts as ≥5 mm in diameter.

CI, confidence interval; BMI, body mass index; MPD, main pancreatic duct

### Long-term follow-up

Long-term follow-up data were obtained for 59.2% (109/184) of participants. The median period between special pancreatic ultrasonography and long-term follow-up was 10.2 years (range, 8.7–11.4 years). New pancreatic findings were detected in two cases (pancreatic cyst, 1; chronic pancreatitis, 1), while no cases of PC were observed.

## Discussion

Although several studies have shown the risk of PC in first-degree relatives and spouses, limited data exist on the risk of MPD dilatation and the development of pancreatic cysts in these individuals [6, 11, 21–23]. In this study, we found that the frequency of ultrasonographic findings of MPD dilatation and pancreatic cysts was significantly higher in spouses of patients with PC than in relatives of patients with PC (33.3% vs. 8.8%), whereas the median age was significantly different (65 vs. 41 years) between both groups because approximately 70% (102/148) of the relative group were children of patients with PC. Multivariate analyses adjusted for participant characteristics revealed that age was an independent risk factor for MPD dilatation and the development of pancreatic cysts. On the other hand, being a spouse was not identified as a risk factor, although spouses tended to be correlated with a higher risk of these ultrasonographic findings.

Several studies have shown the prevalence of unsuspected pancreatic cysts and demonstrated that the frequency of pancreatic cysts increases with age [24–27]. A previous study in patients undergoing magnetic resonance imaging (MRI) for surveillance revealed that the frequency of pancreatic cysts was 1.3% in those aged 40–49 years, 2.6% in those aged 50–59 years, and 3.6% in those aged 60–69 years [26]. Meanwhile, a retrospective analysis using 3-tesla MRI showed that the prevalence of incidental cysts was 5.5% in those aged 40–49 years, 12.1% in those aged 50–59 years, and 19.0% in those aged 60–69 years [27]. In our study, pancreatic cysts were detected in seven participants (4.7%) in the relative group (median age: 41 years) and five participants (13.9%) in the spouse group (median age: 65 years), suggesting that the frequency of pancreatic cysts in this study was similar to that of the corresponding age group in previous studies.

Although MPD dilatation is an independent risk factor for the development of PC [18], there have been few studies on the frequency of MPD dilatation. A previous study using 3-tesla MRI revealed that the frequency of incidental MPD dilatations, defined as an MPD diameter of ≥2.5 mm, was 2.7% [27], while MPD dilatations ≥3 mm in diameter were determined on abdominal ultrasonography in only 0.19% of cases [28]. We previously reported that MPD dilatations ≥2 mm in diameter were observed in 5.03% of the control group using conventional transabdominal ultrasonography (2.44% in 26–50 years of age, 5.35% in 51–75 years of age, and 10.37% in ≥76 years of age) [29]. In contrast, with special pancreatic ultrasonography, MPD dilatation defined as ≥2.5 mm in diameter was detected in seven participants (4.7%) in the relative group and nine participants (25.0%) in the spouse group, suggesting that the frequency of MPD dilatation, especially in the spouse group, is significantly higher than that of the general population reported in previous studies. In the study examining asymptomatic high-risk individuals for PC development, approximately 20% of the participants exhibited mild MPD dilatation, which was significantly associated with neoplastic progression [6]. In contrast, the detailed mechanism of MPD dilatation in high-risk individuals remains to be elucidated. Hyperplastic changes in the epithelium of the pancreatic duct or mucus-producing mechanisms may cause occurrence of PC and affect the slight dilatation of the pancreatic duct [29]. Because individuals with MPD dilatation can be considered a high-risk group for pancreatic diseases [28, 29], further studies focusing on MPD dilatation in individuals with risk factors for PC are required.

This study has some limitations. First, pancreatic cysts and MPD dilatation were evaluated only with special pancreatic ultrasonography. The possibility of missing pancreatic cysts remains because of insufficient observation. However, special pancreatic ultrasonography is a procedure dedicated to observing the pancreas and is a reliable imaging modality to evaluate pancreatic cysts and MPD dilatation with minimal invasiveness. We previously reported that the sensitivity of special pancreatic ultrasonography for detecting pancreatic cysts, confirmed by magnetic resonance cholangiopancreatography, was as high as 92.2% [16]. The capability of transabdominal ultrasonography to detect pancreatic disorders is reduced by obesity [7]. In this study population, the median BMI value was comparatively small (21.6 kg/m^2^). In individuals whose BMI value is high, endoscopic ultrasound and other modalities such as MRI can be alternative imaging modalities to visualize the whole pancreas. Second, there was a significant age gap between the two groups, as approximately 70% of the relative group were children of patients with PC. More recruitment of siblings to the relative group was needed to close this age gap. Third, the risk of developing cancer was not sufficiently evaluated in this study. Because approximately 40% of participants were lost to follow-up, we may have failed to identify the participants who developed PC or other pancreatic diseases.

In conclusion, the frequency of ultrasonographic findings of MPD dilatation and pancreatic cysts was significantly higher in spouses than in first-degree relatives of patients with PC and was strongly influenced by the age gap between the groups. Although the significance of screening for spouses was not clearly presented in this study, it is of note that MPD dilatation was frequently observed among participants, especially in the spouse group, highlighting the need for further studies investigating the frequency of MPD dilatation and its significance, both from pathological and epidemiological perspectives.
